# Assessment of Surgical Tasks Using Neuroimaging Dataset (ASTaUND)

**DOI:** 10.1038/s41597-023-02603-3

**Published:** 2023-10-14

**Authors:** Anil Kamat, Condell Eastmond, Yuanyuan Gao, Arun Nemani, Erim Yanik, Lora Cavuoto, Matthew Hackett, Jack Norfleet, Steven Schwaitzberg, Suvranu De, Xavier Intes

**Affiliations:** 1 Center for Modeling, Simulation, and Imaging for Medicine, Rensselaer Polytechnic Institute, Troy, New York 12180 USA; 2 Boston University Neurophotonics Center, Boston, Massachusetts 02215 USA; 3 Florida A&M University-Florida State University College of Engineering, Tallahassee, FL 32310 USA; 4 Department of Industrial and Systems Engineering, University at Buffalo, Buffalo, NY 14260 USA; 5 University at Buffalo School of Medicine and Biomedical Sciences, Buffalo, NY 14260 USA; 6 U.S. Army Combat Capabilities Development Command – Soldier Center (CCDC SC), Orlando, FL USA

**Keywords:** Neural decoding, Learning and memory

## Abstract

Functional near-infrared spectroscopy (fNIRS) is a neuroimaging tool for studying brain activity in mobile subjects. Open-access fNIRS datasets are limited to simple and/or motion-restricted tasks. Here, we report a fNIRS dataset acquired on mobile subjects performing Fundamentals of Laparoscopic Surgery (FLS) tasks in a laboratory environment. Demonstrating competency in the FLS tasks is a prerequisite for board certification in general surgery in the United States. The ASTaUND data set was acquired over four different studies. We provide the relevant information about the hardware, FLS task execution protocols, and subject demographics to facilitate the use of this open-access data set. We also provide the concurrent FLS scores, a quantitative metric for surgical skill assessment developed by the FLS committee. This data set is expected to support the growing field of assessing surgical skills via neuroimaging data and provide an example of data processing pipeline for use in realistic, non-restrictive environments.

## Background & Summary

fNIRS is a neuroimaging technique that measures the local change in oxyhemoglobin (HbO) and deoxyhemoglobin (HbR) concentration in the brain using different wavelengths of light in the near-infrared spectrum^1^. The relatively high spatial resolution and absence of volume conduction make it preferable to electroencephalography (EEG) for measuring cortical activation in unrestricted tasks. fNIRS probes can be deployed on a low-weight scalp device that facilitates its use in a naturalistic environment^2^ without restricting body movement, unlike gold standard magnetic resonance imaging (MRI). A series of exploratory studies have demonstrated the potential of fNIRS to monitor the cortical activation of subjects while executing diverse surgical tasks^3,4^.

The Fundamentals of Laparoscopic Surgery (FLS) program has been adopted as a prerequisite for board certification in general surgery in the United States^5^. As a part of the FLS program, five psychomotor tasks of increasing complexity must be performed in a trainer box: (i) pegboard transfer, (ii) pattern cutting (PC), (iii) placement of a ligating loop, (iv) suturing with extracorporeal knot tying, and (v) suturing with intracorporeal knot tying. The metric used for manual skill assessment, the FLS score, is computed by an expert proctor for each trial of the surgical task being performed, making it labor-intensive and time-consuming to calculate^6^. As a result, there is motivation to develop a method of predicting the FLS score quickly and without the need for manual assessment. fNIRS is as an ideal device to support evaluation of the FLS tasks as it is portable, noninvasive and nonrestrictive of body movement. It has already been shown to have the potential for assessing the cortical activations associated with surgical task performance^3,4^.

Briefly, fNIRS techniques can be used to reveal different spatiotemporal features of cortical brain activations between surgeons of different levels of proficiency^7–10^. In turn, these distinct cortical signatures can be leveraged to perform skill-level classification, and FLS score prediction. Combining such neurosignatures with a fast and robust predictive model, such as deep learning, can provide new means to support a more efficient training regimen and offer new insights into surgical skill acquisition^11–16^. Moreover, cortical activations can also be used to measure a surgeon’s stress/cognitive load^6,17^, and concentration/attention^16,18^ which can significantly impact patient safety and healthcare economics. Last, fNIRS measurement during the FLS task allows for studying the functional organization of the cortical systems of motor execution and motor learning. Brain connectivity has provided insights into the directed and undirected neural interaction during FLS tasks^15,19–21^.

Beyond monitoring cortical activations, it is also expected that neuromodulation techniques could facilitate the acquisition of complex motor skills. Indeed, studies have shown evidence of enhancing motor skill acquisition by stimulating the brain with weak electric current^4,22–24^. Though the underlying neurophysiology is still poorly understood, combining neuromodulation with neuroimaging techniques may offer the possibility of better understanding these neural interactions^25,26^. Still, limited studies have been done on how modulation affects surgical skill acquisition.

Here, we have conducted a series of studies to collect fNIRS data sets on medical students and/or surgeons performing the FLS PC task. The datasets are organized into four studies: the Expert vs. Novice surgeons’ study (n = 14), the Learning Curve study (n = 13), the short-term transcranial Electrical Stimulation (tES) study, (n = 12) and the long-term tES study (n = 21). The different characteristics of each study along with the associated outcome and technical details are presented in the rest of the paper. We report herein on the release of these fNIRS datasets to support the growing fields of data acquisition and processing in realistic environments, surgical skill assessment and training via neuroimaging data^10^, and data-driven modeling methodologies such as machine learning. This collection of datasets has been utilized in a wide range of studies: (1) classification of the subject’s expertise level (n = 14)^9,27,28^, (2) monitoring surgical skill acquisition (n = 39)^25,27^, (3) benchmarking against and/or prediction the FLS score (n = 14^27^, n = 13^12^), (4) establishing a brain and behavior relationship^29^. While this dataset is comprised of four distinct studies, there is commonality between these studies in the simultaneous recording of fNIRS data with participants performing trials of the FLS PC task, providing a large, labeled dataset for functional neuroimaging for surgical skill assessment.

## Materials and Methods

The Institutional Review Boards of the University at Buffalo, Rensselaer Polytechnic Institute, and Massachusetts General Hospital (MGH) approved the studies. Written consent was obtained from each subject before starting the study. The fNIRS signals were measured simultaneously during the motor task performance by a continuous-wave near-infrared spectrometry covering the prefrontal cortex (PFC), supplementary motor area (SMA), and primary motor cortex (M1).

### Hardware and equipment

The FLS program relies on a physical box trainer to acquire and measure technical skills. The physical simulator consists of an enclosed box in which laparoscopic surgical tools can be inserted through narrow incisions for manipulation. Trainees can use these tools to practice surgical tasks by looking at the real-time video. The PC task used to assess surgical skills was performed in an FLS trainer box.

**Fig. 1:**
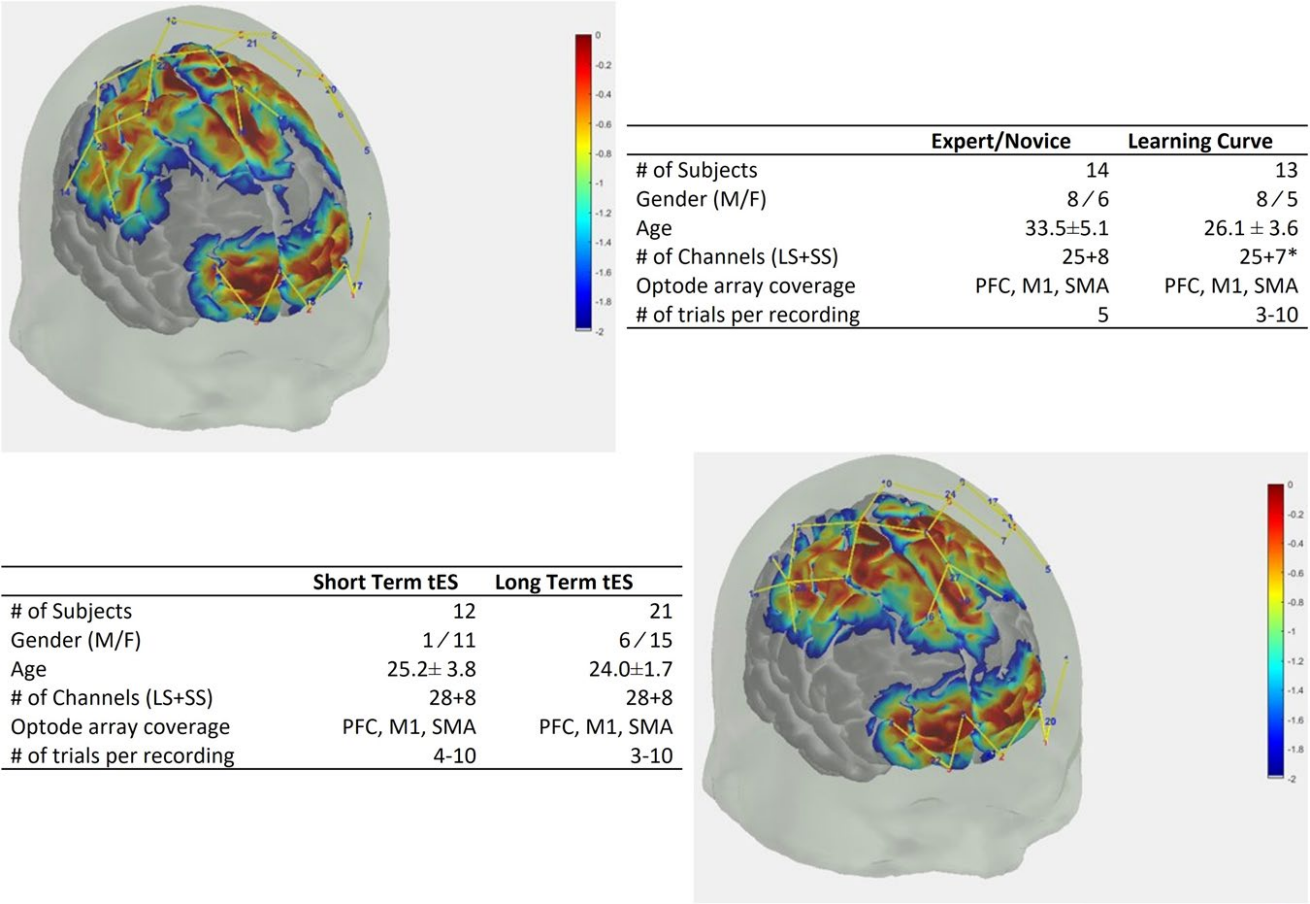
Summary of metadata for each dataset with sensitivity analysis plots of the fNIRS montage used in the Expert/Novice and Learning Curve datasets (top) and transcranial Electrical Stimulation datasets (bottom); LS, Long Separation. SS, Short Separation. PFC, Prefrontal Cortex. M1, Primary Motor Cortex. SMA, Supplementary Motor Area. *Due to hardware issues, data from one short separation channel was not collected in the learning curve data.

For the Expert vs. Novice and Learning Curve studies, we utilized a validated continuous-wave, 32-channel near-infrared spectrometer for this study, which delivered light at 690 nm and 830 nm. (CW6 system, TechEn Inc., MA, USA). The system employed eight long illumination fibers and eight corresponding short-separation detectors coupled to 16 detectors. The long-separation channels comprised all the measurements within a 30–40 mm distance between the source and the detector, and the short-distance channels comprised all the measurements within a ~8 mm distance between the source and the detector. The probe design was assessed using Monte Carlo simulations and was characterized to have high sensitivity to functional changes in the PFC, M1, and SMA (see Fig. 1). To obtain structural guidance and to maintain consistency across subjects with different head sizes while wearing the cap, fNIRS channels were localized with respect to the international 10-5 landmark system.

In the studies which incorporated tES, the commercially available NIRScout (NIRx, Berlin, Germany) was used. This system delivered continuous wave infrared light at 760 nm and 850 nm. The montage used in these experiments was identical to that of the previous montage, with the exception of three additional detectors, which were added to increase sensitivity to the left M1, right M1, and SMA area (as shown in Fig. 1). This resulted in eight sources,19 detectors, and 28 long-separation channels.

The tES was delivered by the StarStim device (StarStim, Neuroelectronics, Spain) with sintered Ag/AgCL pellet electrodes with a diameter of 12 mm and a resulting contact area of 1 cm^2^. One electrode was placed over the left M1 region (C3 on the 10–20 EEG system), and the other was over the right PFC region (Fp2 on the 10–20 EEG system). Transcranial random noise stimulation (tRNS) was delivered at 1 mA, 0.1–650 Hz. Transcranial direct current stimulation (tDCS) started at zero mA and ramped up to 1 mA over the course of 30 seconds, before being ramped back down to zero mA at the end of the stimulation period. The sham stimulation was set at zero current with a 30 second ramp-up to 1 mA and down to zero current at the beginning and the end of the stimulation period to mimic the cutaneous sensation of current changing of tDCS.

Using AtlasViewer^30^, the sensitivities of the probes to changes on the cerebral cortex were assessed using Monte Carlo simulations of photon propagation^31^. The resulting sensitivity maps for each of the probes described above were projected to the Atlas’ cortex and are shown in Fig. 1.

### Surgical task

The PC task is one of the five psychomotor tasks in the FLS program, where the goal is to use laparoscopic tools to cut a marked piece of gauze as quickly and as accurately as possible. The subjects must operate in a volume-constrained 3D environment while looking at the 2D monitor screen for visual feedback. All participants were instructed on how to perform the task with standardized verbal instructions (or video) indicating the goal of the task and rules for task completion. All the experiments were conducted in adequate lighting.

Task performance was measured following the FLS guidelines^32^. The raw score was calculated based on completion time and errors^33^, with a higher score indicating better performance. Proficient performance is considered as completion within 98 seconds with no deviation outside the drawn circles (see https://www.flsprogram.org/technical-skills-training-curriculum/).

### Subjects and study design

**Fig. 2:**
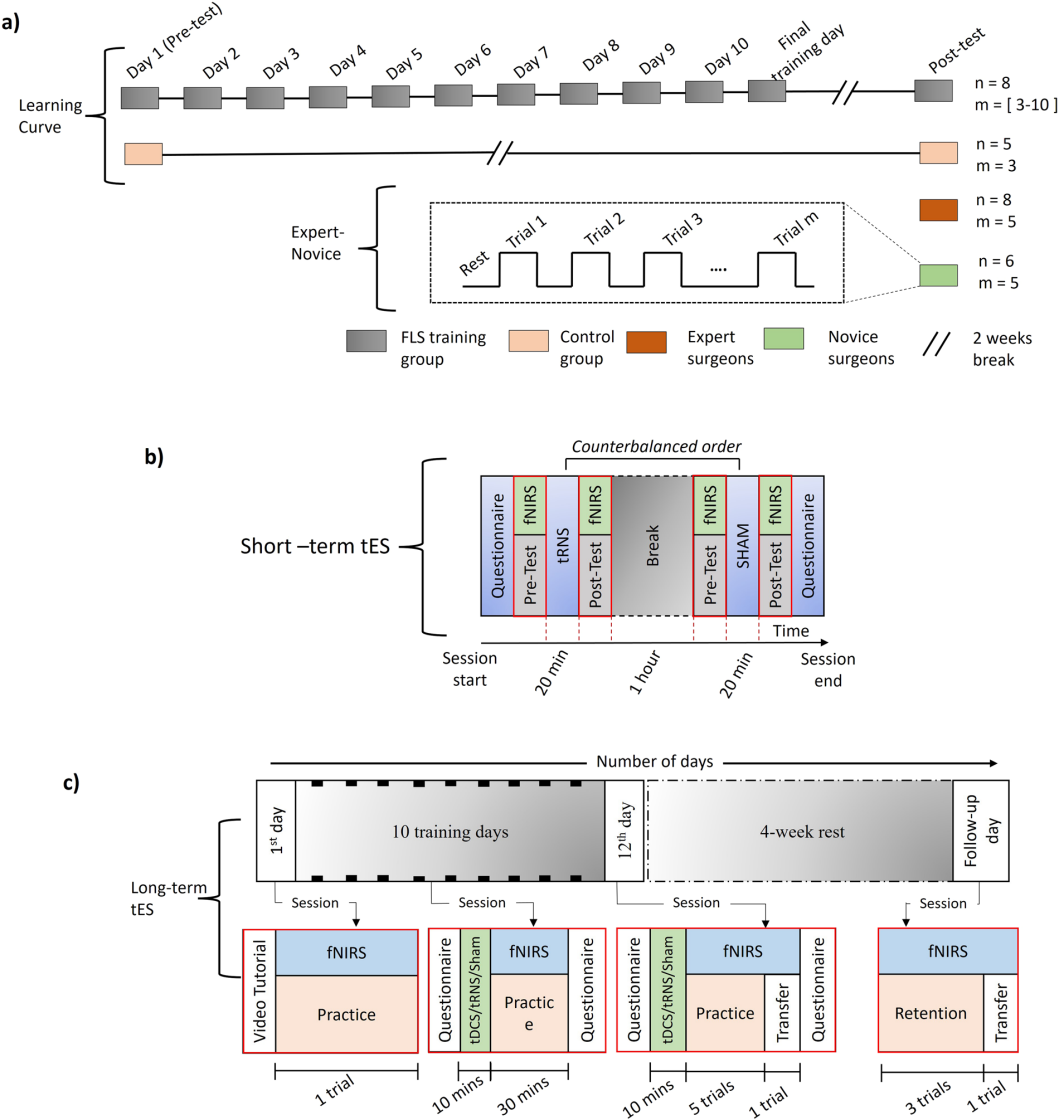
(**a**) Schematic Outlining Cohort and Study Design: The learning curve study design is depicted in a gray box chain format, where each box represents a day of the study. The untrained control group (orange) performed the task on the first day and again on the post-test day. The block design of expert and novice surgeons is shown in red and green boxes respectively. Note: ‘‘n’’ is number of subjects and ‘‘m’’ is number of trails performed by each subject. (**b**) Short-term tES Study Block Design: The vertical red box labeled “Pre/Post-test and fNIRS” indicates that these components were conducted simultaneously. The time indicated at the bottom of the block represents the duration spent on each respective component. (**c**) block design long-term tES study: Each small black box represents one training day. The blocks on the bottom row (red boxes) indicate the specific procedures carried out on each session on the respective day.

The experiment consisted of a block design (Fig. 2a–c)) of rest and stimulus period (i.e., PC task). PC was performed until completion or stopped after five minutes. Then a rest period of approximately thirty seconds in tES studies or one minute in the Expert vs. Novice and Learning Curve studies was observed to allow the brain activation to reach back to the baseline period. The cycle of cutting tasks and rest periods was repeated multiple times for each participant.

### Expert vs. novice study

The population of this study was split into a novice group (n = 6, 1^st^-3^rd^ year residents with a mean age of 31.33 ± 1.2 years) and an expert group (n = 8, 4^th^ and 5th-year residents and attending surgeons with a mean age of 35.1 ± 6.4 years). Each subject performed the PC task 5 times, with a 1-minute rest period between each trial (see Fig. 2a). The aim of this study was to determine if functional neuroimaging could distinguish between skilled and unskilled surgical performance. In this study, all subjects performed 5 trials in a single experimental session.

### Learning curve study

Recruited subjects were split into FLS training and a control group with no training. The sample population included novices with no prior FLS training (n = 8 training, n = 5 control, 1^st^ – 3^rd^-year residents with a mean age of 26.1 ± 3.6 years). Note that both groups were independent, i.e., each subject belonged to only one group. The untrained control group performed three trials on the physical simulator (i.e., FLS box trainer) and three on a virtual reality-based PC simulator (Virtual Basic Laparoscopic Skill Trainer (VBLaST) (virtual simulator data is not published here), validated in^9^) on the first day. After a 2-week period, the control group performed three FLS trials as part of the final skill retention day without undergoing any laparoscopic skills training. The FLS training groups were instructed to complete up to 10 trials per day for twelve consecutive days on each group’s respective simulator (see Fig. 2a). Following twelve days of training, each group waited two weeks without undergoing any laparoscopic training before performing three FLS trials as part of the final skill retention day and a transfer task, which involved cutting a similarly sized circle out of a piece of *ex vivo* human cadaveric peritoneal tissue. Unlike the previous study, this study consisted entirely of unskilled surgical trainees. The goal of this study was to determine if surgical skill acquisition could be monitored via functional neuroimaging, and monitors performance of subjects as they train to reach proficiency in the PC task.

### Short-term tES study

Twelve novice medical students (n = 12 mean age, 25.2 ± 3.8 years) were recruited. Before the experiment started, the participants watched a video to learn how to perform the task and then performed 10 trials of PC while fNIRS data was recorded to become familiar with the box trainer and fNIRS recording cap. All subjects then completed two sequential testing blocks, one of tRNS and one of sham stimulation (order counterbalanced), with an hour of rest in between. Each of these testing blocks consisted of four trials before stimulation, 20 minutes of sham or tRNS stimulation, and then four trials after stimulation (see Fig. 2b). fNIRS data were recorded during each of these PC trials. The aim of this study was to determine whether transcranial electrical stimulation had an impact on subjects’ performance during surgical skill acquisition. Like the previous study, this study was composed entirely of unskilled surgical trainees but took place over one experimental session per subject, similar to the expert vs novice study.

### Long-term tES study

The population distributions for this study were as follows: tDCS (n = 7, mean age 24 ± 3), tRNS (n = 7, mean age 23 ± 1), and Sham (n = 7, mean age 23 ± 1). The participants underwent 12 visits on 12 consecutive days and one visit as a follow-up four weeks later. From day-2 to day-11, the subjects underwent 10 minutes of stimulation (tDCS/tRNS/Sham according to their group assignment) and then 30 minutes of practice while fNIRS data was collected. On the 12^th^ day, after five trials of the P.C. task practice, they performed a transfer task, where they removed a marked peritoneal layer of an *ex vivo* porcine abdominal wall. Four weeks after the completion of the training, subjects returned for a follow-up visit, during which they performed the same PC task three times, along with one transfer task, to measure their skill retention and transfer skill retention (see Fig. 2c for the block design). Building off of the previous study, this study used functional neuroimaging to monitor surgical skill acquisition over the course of multiple training days and a follow-up retention day after a period without training, similar to the learning curve study. The difference between this study and the learning curve study is that this study aimed to compare surgical skill acquisition across different transcranial electrical stimulation conditions, tDCS, tRNS and sham stimulation.

### Data processing

**Fig. 3:**
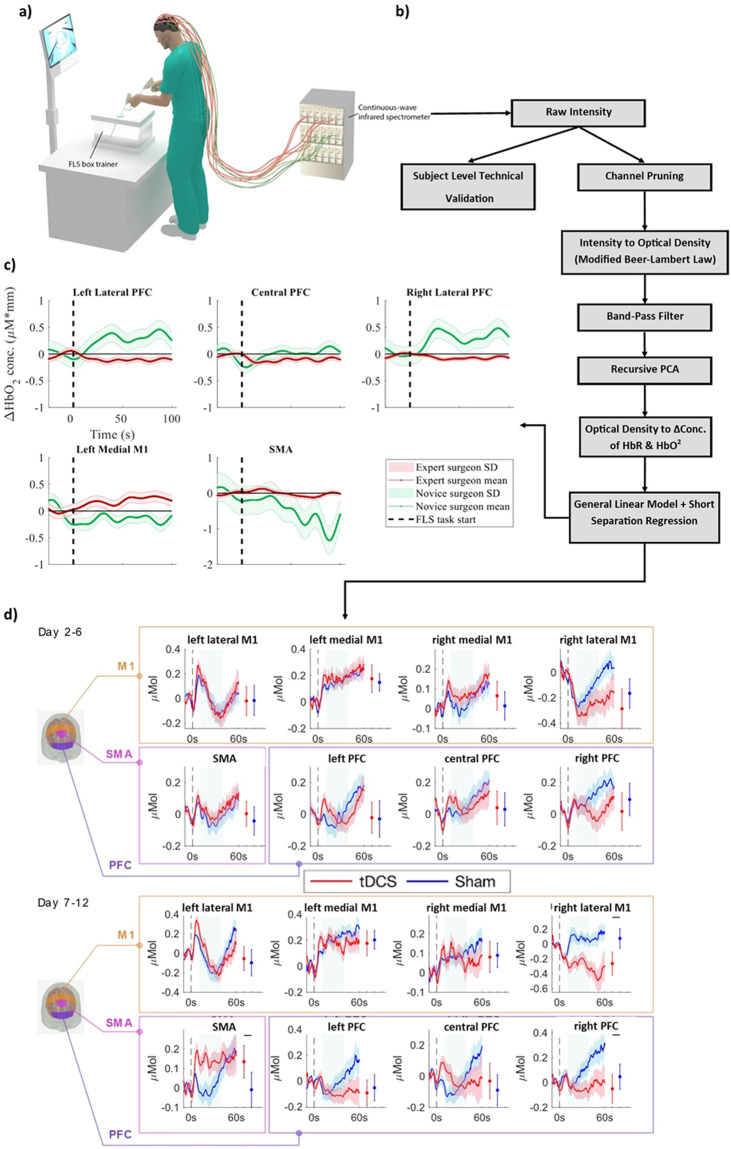
(**a**) Illustration of surgical task performance with simultaneous fNIRS data collection. (**b**) Diagram visualizing the pipeline for data processing and technical validation (**c**) The HRF of each of the sampled cortex regions, averaged across the Expert/Novice dataset participants. Adapted from ref. ^28^. (**d**) The HRF of each of the sampled cortex regions was averaged across the participants of the Long-Term tES dataset. Adapted from ref. ^17^.

In order to promote the reproducibility of results and provide an example pipeline for those unfamiliar with fNIRS data processing but interested in this dataset, we describe the data processing pipeline used in our analysis, however, we only provide the raw data files. Data processing was completed using the open-source software HOMER2. Due to different hardware being used for collecting the datasets, separate processing steps and parameters were used for the datasets collected with each imaging system. Here, we will give an example of the processing pipeline used in the studies which were collected on the TechEn system (the Expert vs. Novice and Learning Curve datasets). First, channels with signal quality outside 80 dB to 140 dB were excluded. Next, the remaining raw optical signals (wavelengths at 690 nm and 830 nm) were converted into optical density. Then a band-pass filter of 0–0.50 Hz was used to denoise the signal. As fNIRS is susceptible to motion artifacts, they need to be detected and removed/corrected before analysis. Motion artifacts and systemic physiology interference were corrected using recursive principal component analysis and low-pass filters. Using the modified Beer-Lambert law with partial path-length factors of 6.4 (690 nm) and 5.8 (830 nm), the changes in concentrations of oxy and deoxyhemoglobin were extracted. Finally, the most correlated short-distance channels are regressed from the long-distance channels to remove any interference from superficial layers using a consecutive sequence of Gaussian basis functions via ordinary least squares to create the hemodynamic response function (HRF). Figure 3 displays an example of this pipeline and the resulting HRFs achieved using two of the datasets presented, the expert/novice and long-term tES studies. The details of the preprocessing pipeline for all the studies can be found in the excel sheet in the data folder.

## Data Records

Data is publicly available and can be accessed from the Figshare^34^. Each dataset is uploaded in a separate directory and consists of the raw data in the form of Shared Near Infrared File Format (.snirf) files (see https://github.com/fNIRS/snirf) and in Brain Imaging Data Structure (BIDS) (see https://bids.neuroimaging.io/) format. A Microsoft Excel (.xlsx) spreadsheet contains the metadata for each dataset, the subject demographics, and the FLS score corresponding to each trial. Each dataset is organized in a similar structure, with subdirectory in order of experimental condition (if applicable), subject, and finally, each recording. Each recording is labeled in the following pattern: subject identifier-training day/condition-number of trials in the recording.

Additionally, in the repository (Figshare^34^), we have included a separate metadata sheet (in CSV format) and a corresponding readme file for each experiment. These files provide detailed information about various aspects of the study, including demography, safety assessments, performance scores, variables, parameters used, and fNIRS file names for easy cross-referencing. The metadata sheets and readme files serve as comprehensive references for understanding the experimental data and its associated components.

## Technical Validation

**Fig. 4:**
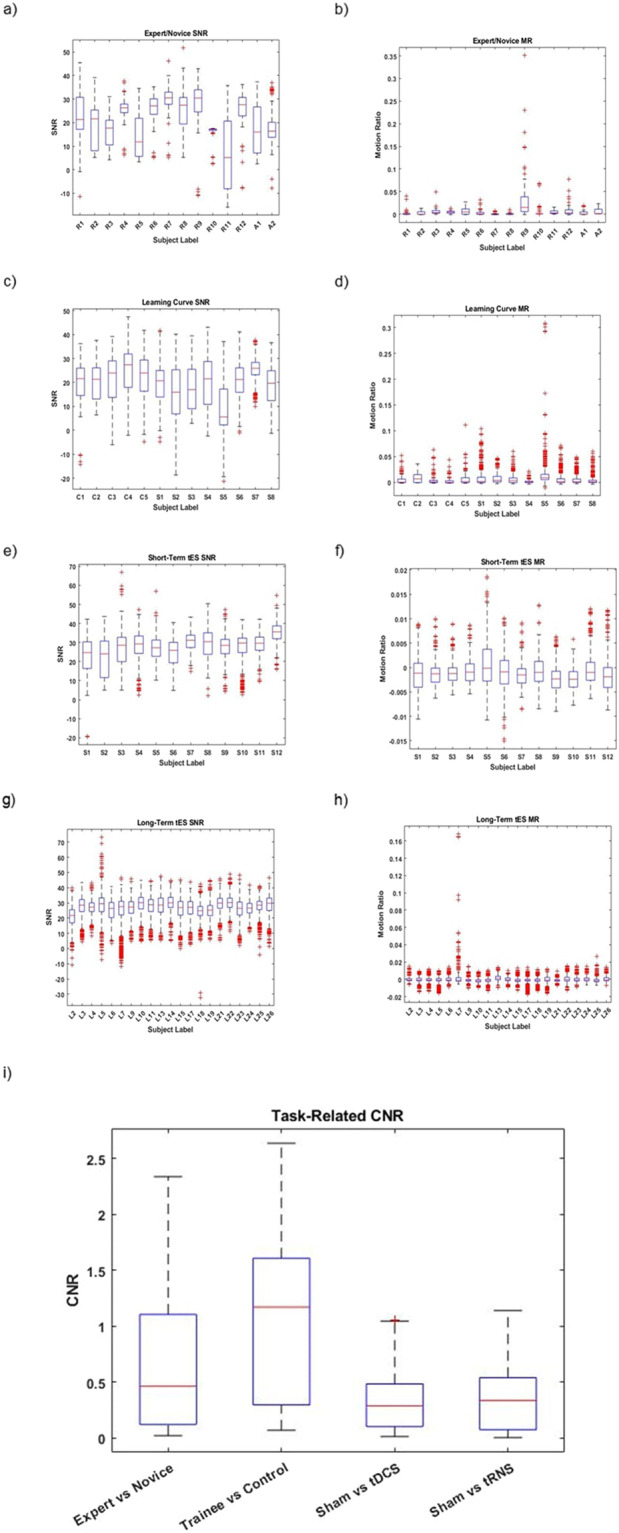
(**a,c,e,g**) Subject-wise (across all channels and trials for each subject) signal to noise ratio calculations for the Expert vs. Novice, Learning Curve, Short-Term tES and Long-Term tES studies respectively. (**b,d,f,h**) Subject-Wise motion ratio calculations for the Expert vs. Novice, Learning Curve, Short-Term tES and Long-Term tES studies respectively. (**i**) Task-related contrast to noise ratio for Expert vs Novice, Trainee vs Control, Sham vs tDCS and Sham vs tRNS populations.

A quality assessment of signals collected is necessary to avoid spurious interpretation of the results and to ensure reproducibility^22^. Here, we present a quality assessment of the collected signals. To give an accurate assessment of the quality of the data, no channels have been excluded from the analysis provided here and no denoising methods have been used prior to analysis. Because fNIRS collects signals noninvasively, many physiological signals which originate from other parts of the body, such as cardiac rhythm, blood pressure fluctuation, and respiratory rate, are picked up by the fNIRS device, and it becomes essential to validate the measured signals. Furthermore, due to the non-invasive method of fNIRS, the decoupling of the optodes from the scalp produces motion artifacts which are considered confounding signals^35^. To assess the quality of the datasets, we provide the signal-to-noise ratio and motion ratio here. The signal-to-noise ratio (SNR) is a method of assessing the signal quality and is calculated as $SNR=20\left(\frac{\mu }{\sigma }\right)$, where µ is the mean of the signal and σ is the standard deviation of the signal^36^. Signals with high SNR are desirable as they are indicative of higher signal quality. Further, most of the conventional statistical testing methods require balanced (equal) SNR as they depend on equality of variance^24^. In this study, SNR was calculated for each channel using the entire time series for each recording (see Fig. 4a,c,e,g). The motion ratio is calculated as the ratio between the total time of segments in the data considered confounded by motion artifacts to the total acquisition time^36^ (see Fig. 4b,d,f,h). The duration of motion artifacts was identified using the Homer3 function *hmrR_MotionArtifactByChannel* with parameters suggested in^36^. While ideally, the data would be free of noise and motion artifacts; we can see from Fig. 4 that motion artifacts and lower SNR values affect some channels. It is not unexpected that the data quality here is impacted due to the amount of motion and time required to complete the PC task in a natural and unrestricted environment. Furthermore, it is important that task-related hemodynamic activity is present in the data. To ensure this, we calculate the Contrast-to-Noise Ratio (CNR) as defined by^37^ between two comparable populations. First, we calculate the group mean HRFs for each population using the processing pipelines specified for each study in Homer3 (excluding channel pruning). The populations being compared are the expert and novice groups from the Expert vs. Novice study, the Trainee vs. Control group for the learning curve study, the sham condition vs. tDCS and sham condition vs tRNS from the Long-Term tES study. Once group mean HRFs are calculated we calculate the channel-wise CNR between populations by dividing the absolute value of difference of mean between each HRF by the square root of the sum of the variances of the two HRFs being compared. Figure 4i shows the resulting values for each comparison. For more in depth analysis of the population-level differences and scientific conclusions, please refer to the following:^12,25,27^.

## Data Availability

The repository of preprocessing and quality assessment codes for FLS learning curve study, expert-novice, and neuro-modulation studies have been made public and can be assessed at Figshare^34^.
